# Ischemic Risks Induced by Larger Orthodontic Forces on Dental Pulp and Neuro-Vascular Bundle in Reduced Periodontium

**DOI:** 10.3390/jcm13226698

**Published:** 2024-11-07

**Authors:** Radu-Andrei Moga, Cristian Doru Olteanu, Ada Gabriela Delean

**Affiliations:** 1 Department of Cariology, Endodontics and Oral Pathology, School of Dental Medicine, University of Medicine and Pharmacy Iuliu Hatieganu, Str. Motilor 33, 400001 Cluj-Napoca, Romania; ada.delean@umfcluj.ro; 2 Department of Orthodontics, School of Dental Medicine, University of Medicine and Pharmacy Iuliu Hatieganu, Str. Avram Iancu 31, 400083 Cluj-Napoca, Romania

**Keywords:** dental pulp, neuro-vascular bundle, ischemic risks, periodontal breakdown, orthodontic force, finite elements analysis, orthodontic movements

## Abstract

**Background/Objectives:** There are few data about the ischemic risks induced by the large orthodontic forces during periodontal breakdown in dental pulp and neuro-vascular bundle (NVB) and none on the individual tissular stress distribution, despite their great importance for orthodontic treatment planning. Our aim was to assess, by a numerical analysis, the biomechanical behavior of dental pulp and the NVB during a simulated horizontal periodontal breakdown (1–8 mm), under 2–4 N of applied orthodontic forces and five movements (rotation, translation, tipping, intrusion, and extrusion). Additionally, the ischemic and degenerative-resorptive risks were assessed. **Methods:** The analysis involved 72 3D models of nine patients, totaling 720 simulations. The models were CBCT-based, having the second lower premolar and surrounding periodontium, and they suffered 1 mm of gradual horizontal periodontal breakdown (up to 8 mm loss). **Results:** Both forces displayed a similar qualitative stress distribution in all five movements, but with a quantitative increase (doubling of stress amounts for 4 N when compared with 2 N). The highest amounts of stress were displayed at 8 mm of periodontal loss, which is lower than the 16 KPa of the maximum hydrostatic pressure. The NVB stress was higher than the pulpal stress. Rotation was the most stressful, closely followed by tipping, intrusion, and extrusion. **Conclusions:** A total of 4 N of applied force seems to not induce any ischemic or degenerative-resorptive risks for healthy intact teeth, in up to 8 mm of periodontal breakdown. Intrusion and extrusion determined the highest visible tissular deformation in the NVB, with potential ischemic and resorptive-generative risks for previously traumatized/injured teeth (i.e., occlusal trauma). Rotation and translation (in particular) showed the highest coronal and radicular pulpal stress with potential ischemic and resorptive-generative risks for previously injured/traumatized dental pulp (i.e., direct-indirect pulp capping). It seems that 4 mm of periodontal breakdown could signal a clinical stress increase with potential ischemic and degenerative-resorptive risks for the previously traumatized/injured tissues.

## 1. Introduction

One of the clinical issues in the orthodontic treatment is related to the evaluation of ischemic and degenerative-resorptive risks induced by orthodontic forces over the dental pulp and its neuro-vascular bundle (NVB) of healthy intact and/or previously traumatized/injured teeth [1,2,3,4,5,6,7,8]. Since the orthodontic treatment is performed on patients with various levels of bone loss (due to the periodontal breakdown process, more prevalent in younger patients), the risk evaluation becomes even more important clinically [9,10], especially in the context of previously traumatized/injured tissues [1,2,3,4,5,6,7].

There are few studies (in vivo [4,5,6,7,8] and numerical [2,9,10]) regarding the impact of light orthodontic forces (of around 1 N) over the dental pulp in intact periodontium (with various contradictory reports) [9,10]. However, none was focused on the impact of pulp and its NVB in reduced periodontium (except our previous studies [9,10]). It must be emphasized that clinical in vivo studies provide general data about the entire dental tissues, while the individual assessment of each tissular component is performed only through numerical analysis [9,10,11].

Another issue is related to the employment of larger orthodontic forces (2–4 N) in both intact and reduced periodontium and their effects on pulp and its NVB (no such reports were found in the current body of research). There are numerical reports (with various accuracy) on the biomechanical behavior of the periodontal ligament (PDL) in intact periodontium that can be correlated and indirectly used for data regarding the NVB’s behavior [12,13,14,15,16]. It must be emphasized that the NVB is held in the apical third of the PDL and a stress evaluation of this region can provide some data the NVB [3,9,17]. However, since the NVB is an extremely small and complex anatomical structure, none of the previous numerical studies [12,13,14,15,16] reconstructed a PDL apical third with this structure. Thus, the only direct numerical correlations can only be performed with our previous studies, which employed similar methodology as herein [9,10,17,18,19,20,21,22].

For evaluating the ischemic and degenerative-resorptive risks, the anatomical functionality and integrity of the tissues [8,23,24] must also be considered when planning the orthodontic treatment (especially with brackets). The dental tissues have a very good adaptability due to their absorption-dissipation ability (described and quantified only by our previous numerical reports [9,10,17,18,19,20,21,22]), showing little damage if a higher amount of orthodontic force was applied for limited periods of time (backed by both clinical and numerical studies) [2,4,5,6,7,8,9,10]. However, if the tissues were previously traumatized/injured [4,5,7,23,25] (e.g., occlusal trauma particularly affecting the NVB) and/or the tooth had suffered from dental decay [26,27,28,29,30,31,32,33,34,35,36] (e.g., direct-indirect pulp caping particularly affecting coronal and radicular pulp [35]), the reactivity is significantly diminished as well as the adaptability to higher amounts of stress (backed by clinical and numerical studies [4,5,6,7,23,24,25,36,37,38,39,40,41,42]). Both these above-mentioned situations are not clinically visible (and quite clinically frequent), while their consequences are seen only during orthodontic treatment, when it is too late (irreversible mechanisms have already started) [4,5,6,7,25,26,27,28,29,30,31,32,33,34,35,36,37,38,39,40,41,42]. The periodontal breakdown also has an impact through the changes in the tissular biomechanical behavior in the above-mentioned circumstances [3,9,10,17,18,19,20,21,22]. Nevertheless, by numerically evaluating these tissues carefully, the ischemic and resorptive risks could be anticipated and evaluated [3,9,17].

The dental pulp and the NVB’s circulatory vessels hold as physiological maximum hydrostatic pressure/MHP of 16–22 KPa [3,12,13,15,16], preventing their collapse [3,5,6,7,9,17]. If this pressure is exceeded for prolonged periods of time, the induced circulatory disturbances will exceed the local tissular adaptability and induce ischemia leading to degenerative-resorptive processes [1,2,3]. The orthodontic movement is triggered by these above-mentioned local circulatory disturbances [8,9,43], but the amount of the optimal applied orthodontic force is still a subject of controversy. The clinical practice and theory states that are the safest in intact periodontium are the light orthodontic forces (around 1 N) [9,10,18,19,44], while other reports suggest different optimal higher forces [8,43,45,46]. Nevertheless, there are many reports that contradict clinical knowledge [9,10,17,18,19,44], reporting larger optimal orthodontic forces (2–6 N) [12,13,14]. Debatable is also the amount of force applied in reduced periodontium, since the bone loss fundamentally changes the tissular biomechanical behavior (as proven by our previous numerical reports [9,10,17,18,19,20,21,22]). Additionally, more confusing are the reports about the poor quality of multiple in vivo studies [37,43,45]. Thus, the confusion related to the above-mentioned issues results in the need for more numerical studies focused on the biomechanical behavior of each dental tissular component subjected to various amounts of loads and various bone loss levels.

Our previous numerical reports [9,10,17,18,19,20,21,22]) regarding the use of light orthodontic forces on healthy intact teeth and during periodontal breakdown showed a direct correlation regarding the level of bone loss and stress increase in both pulp and the NVB (the only numerical reports available regarding this issue) but with no ischemic risks.

Numerical analysis is the only available method to individually study each tissular component of the dental tissues [9,10,11,17,18,19,20,21,22]. The numerical/finite elements method is an engineering field method with extremely high accuracy employing a 3D numerical model of the analyzed structure, providing both qualitative (color-coded stress display) and quantitative (amount of stress) results [9,10,11,17,18,19,20,21,22]. Specific to this method is the employment of a single model that can be easily modified, while its analyzed conditions (boundary assumptions) can be rapidly changed [9,10,11,17,18,19,20,21,22].

The numerical study method has been imported by the dentistry field in the past two decades, with significant accuracy issues [4,5,6,7,11,12,13,15,16,37,47,48,49,50,51,52,53,54,55,56,57,58,59,60,61], since the mandatory conditions (boundary assumptions) of the engineering field were not fulfilled [9,10,11,17,18,19,20,21,22]. Thus, the dental numerical studies often provide contradictory reports (both among themselves and in relation to clinical realities) [4,5,6,7,11,12,13,15,16,37,47,48,49,50,51,52,53,54,55,56,57,58,59,60,61,62], transforming this valuable method, recognized in the engineering field for its accuracy, into a marginal mistrusted method [9,10,17,18,19,20,21,22]. Numerical studies must always be correlated and validated with known clinical data, the tissular biomechanical behavior, as well as other reports [9,10,17,18,19,20,21,22].

Nevertheless, our stepwise study project (clinical protocol 158/2 April 2018) identified the main problems related to the accuracy issues and provided a methodology that ensures the same accuracy as that in the engineering field [9,10,17,18,19,20,21,22]. It must be emphasized that our study is the only one to address these above-mentioned issues and study the dental tissues in both intact and reduced periodontium [9,10,17,18,19,20,21,22].

The aims of the report herein were the following: (a) the biomechanical behavior assessment of the dental pulp and the NVB under 2 and 4 N during orthodontic movements in the context of a gradual horizontal periodontal breakdown (1–8 mm loss); (b) the assessment of ischemic and degenerative-resorptive risks for the pulp and the NVB in the context of the horizontal periodontal breakdown process correlated with physiological maximum hydrostatic pressure.

## 2. Materials and Methods

The numerical analysis herein is part of an ongoing study (clinical protocol 158/2 April 2018), stepwise conducted regarding the biomechanical behavior of dental tissues subjected to orthodontic forces and movements in various levels of bone loss [9,10,17,18,19,20,21,22].

This study focused on the study of the biomechanical behavior of dental pulp and NVB under larger orthodontic forces during a gradual horizontal periodontal breakdown.

The sample size was nine (nine patients, four males/five females, mean age 29.81 ± 1.45) as in our above-mentioned studies, nine times higher than the sample size of the one specific to this method used by recent dental studies [10,11,12,13,17,18,55,56,57,59]. The study included 72 3D models of the second lower premolar from the nine patients and totaled 720 numerical simulations.

The inclusion criteria were as follows: no missing teeth in the region of interest, no malposition of the 2nd lower premolars, intact premolars free of any dental treatments (endodontic/filling/crown), up to 1–2 mm bone loss, healthy periodontium, orthodontic treatment indication, and proper oral hygiene. The exclusion criteria were as follows: particular root geometry (e.g., non-fused double root, angulated root, extreme curvature), abnormal crown shape with multiple cusps, temporary teeth, abnormal root surface defects (e.g., external root resorption) and bone defects (radiologically visible bone defects), abnormal pulp chamber and root canals (e.g., internal resorption), more than 2 mm bone loss, and poor oral hygiene with visible signs of inflammation.

The radiological image was acquired using a ProMax 3DS Cone Beam Computed Tomography (Planmeca, Helsinki, Finland) and held a voxel size of 0.075 mm. The premolar region was chosen since there is limited research on this region (most reports concerned the incisors and molars) despite its active part in the oral functional activity and occlusal lateral support.

**Figure 1:**
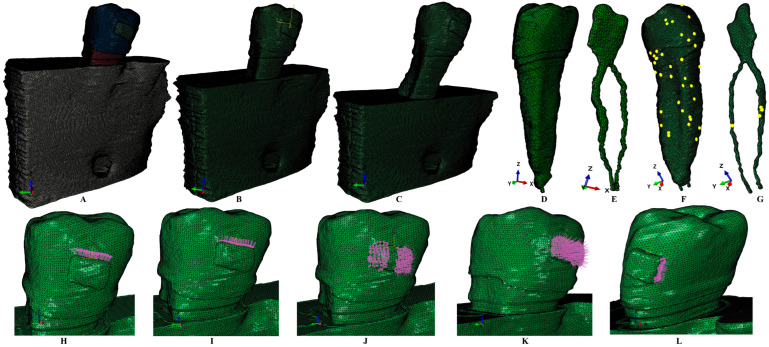
Mesh model (of one of the nine 3D models): (**A**) 2nd lower right premolar model with 1 mm reduced periodontium, (**B**) 2nd lower right premolar model with 4 mm reduced periodontium, (**C**) 2nd lower right premolar model with 8 mm reduced periodontium, (**D**) 2nd lower premolar with dental pulp and NVB, (**E**) dental pulp and NVB, (**F**) 2nd lower premolar with mesh elements warnings, (**G**) dental pulp and NVB mesh with elements warnings; applied vectors: (**H**) extrusion, (**I**) intrusion, (**J**) rotation, (**K**) tipping, (**L**) translation.

**Figure 2:**
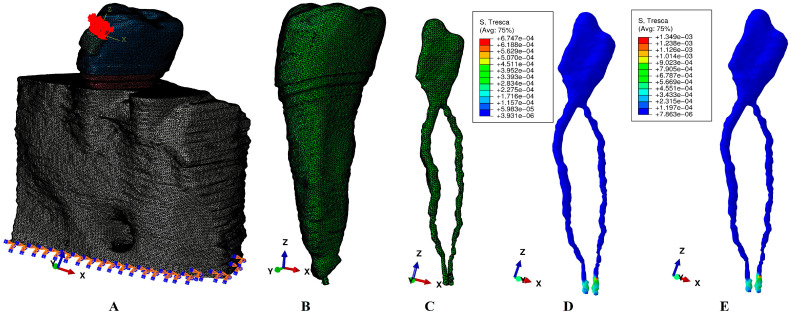
Timeline of the study: (**A**) 1 mm periodontal loss model with extrusion load, (**B**) mesh of the 2nd lower premolar with pulp and NVB, (**C**) mesh of dental pulp and NVB, (**D**) 2 N of extrusion color-coded stress distribution, (**E**) 4 N of extrusion color-coded stress distribution.

The radiological images displayed the investigated tissues (extremely small and complex) as various shades of gray. The tissular reconstruction process was manually performed using the AMIRA 5.4.0 version (Visage Imaging Inc., Andover, MA, USA). During this phase, each tissular component (enamel, dentin, dental pulp, neuro-vascular bundle/NVB, cortical and trabecular bone, and periodontal ligament/PDL) was identified and then selected. The result was an anatomically accurate 3D model of the investigated tissues (Figure 1 and Figure 2). The PDL had a physiological variable thickness of 0.15–0.22 mm and was held in the apical third the NVB. A clear separation between dentin and cement was not possible, thus, given their similar physical properties (Table 1), cement was reconstructed as dentin.

The alveolar socket of the neighboring teeth was filled with trabecular and cortical bone. The missing bone and PDL were also reconstructed to obtain 3D models with no bone loss. A stainless-steel bracket base (to avoid any potential interference) was also reconstructed on the vestibular side of the crown. Each intact periodontium model was subjected to a gradual horizontal periodontal loss of 1 mm (up to 8 mm of loss). Thus, the study was performed on seventy-two models with a bone loss from 1 to 8 mm (eight models derived from each of the nine models with intact periodontium).

The 3D mesh models held 5.06–6.05 million C3D4 tetrahedral elements, 0.97–1.07 million nodes, and an extremely small global element size of 0.08–0.116 mm (Figure 1). The mesh testing processes revealed no element errors and only element warnings (but in an extremely small number). Thus, the highest number of element warnings in one of the intact periodontium models for the tooth (Figure 1) was 39 (0.00589%) warnings for a total of 661,137 elements, while the pulp and the NVB showed only 4 warnings (0.0158%) for a total of 25,252 elements. All the algorithm-based internal control processes were successfully passed since there was quasi-continuity in all essential areas, and the element warnings were held in non-essential areas.

The seventy-two models were then imported in ABAQUS 6.13-1(Dassault Systèmes Simulia Corp., Maastricht, The Netherlands) for numerical analysis. The boundary assumptions were the following: models with zero displacements, perfectly bonded interfaces, isotropy, homogeneity, and linear elasticity, as well as the Tresca failure criteria (maximum shear stress). All these were proven to provide accurate results based on our team’s previous reports [9,10,17,18,19,20,21,22]. Five of the most used orthodontic movements (extrusion, intrusion, rotation, tipping, and translation) under 2 and 4 N were simulated.

The numerical analysis is displayed in pulp and NVB color-coded projections of the stress distribution (i.e., red–orange high, yellow–green moderate, blue low) associated with quantitative amounts of stress. These were then correlated with the physiological maximum hydrostatic pressure of 16–22 KPa (present at this level), as well as with the results of the available reports.

## 3. Results

**Figure 3:**
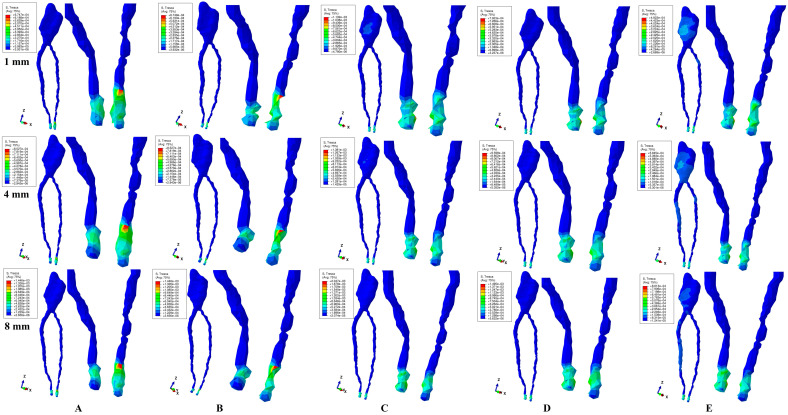
Comparative stress distribution for 2 N applied force (of one of the nine 3D models) in 1 mm, 4 mm and 8 mm horizontal periodontal breakdown: (**A**) extrusion, (**B**) intrusion, (**C**) rotation, (**D**) tipping, (**E**) translation.

**Figure 4:**
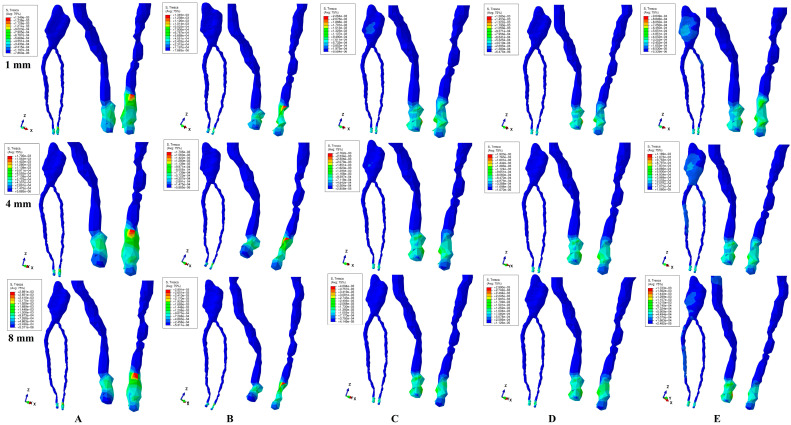
Comparative stress distribution for 4 N applied force (of one of the nine 3D models) in 1 mm, 4 mm, and 8 mm horizontal periodontal breakdowns: (**A**) extrusion, (**B**) intrusion, (**C**) rotation, (**D**) tipping, (**E**) translation.

All quantitative amounts of stresses displayed by the 2–4 N of applied forces in the dental pulp and the NVB were under 16 KPa of the MHP (Figure 3 and Figure 4, Table 2) during the entire horizontal periodontal breakdown simulation.

The numerical simulations showed that the highest amounts of stress were displayed at the NVB level by all five movements, independent of the amount of applied force (Table 2 and Figure 3 and Figure 4) and during the entire bone loss simulation. Based on the above-mentioned, circulatory disturbances are present at this level during orthodontic movements and are strictly correlated with the periodontal level, increasing with bone loss, but with no significant ischemic or resorptive-degenerative risks for intact teeth.

The dental pulp (both coronal and root-canal) displayed only limited amounts of stress, with no expected ischemic or resorptive-degenerative risks in intact teeth (Table 2 and Figure 3 and Figure 4).

Qualitatively, both applied forces displayed similar stress distributions (Figure 3 and Figure 4), with quantitative stress values doubling for 4 N force when compared with the 2 N force (Table 2), for all five movements and horizontal periodontal breakdowns. Thus, 2–4 N of force seem to be relatively safe on up to 8 mm of bone loss for both the dental pulp and the NVB, for intact teeth with no previous periodontal injuries and/or trauma.

The rotation was the most stressful movement during the entire periodontal breakdown simulation (Table 2—NVB displaying 0.95 KPa and 2.05 KPa for 1 and 8 mm of bone loss, respectively, 8–16 times lower than the MHP). Tipping was slightly less stressful (than rotation), closely followed by intrusion and extrusion. The translation was the least stressful among all five movements in all periodontal breakdown simulations.

Quantitatively, NVB and dental pulp stress increases for both forces, which seems to be strictly corelated with the bone loss level for all five movements, thus, at 8 mm of loss, it was visibly displaying a doubling of stress amounts when compared with 1 mm of loss (Table 2).

Qualitatively, the color-coded stress display (Figure 3 and Figure 4) shows localized high intensity red–orange areas at the NVB level for all movements, with intrusion and extrusion being the most extensive. The above-mentioned correlated with the visibly higher tissular deformations (Figure 3A,B and Figure 4A,B), which could lead to localized circulatory disturbances with ischemic and degenerative-resorptive risks (increasing along with bone loss) in teeth with previously traumatized and/or injured periodontium. Thus, higher tissular deformations are visible after 4 mm of bone loss, and when the stress increases it doubles its values, making intrusion and extrusion prone to ischemic and degenerative-resorptive risks (more than the other three movements), especially for the previously traumatized/injured teeth.

Lower intensity blue areas were visible during rotation and translation movements (for both forces) in the coronal pulp on the vestibular, mesial, and distal sides, especially for 1 mm of bone loss, signaling potential ischemic and degenerative-resorptive risks for the dental pulp that previously suffered various dental treatments (i.e., direct and indirect pulp capping). The stress display areas decreased with bone loss. Thus, at 4 mm of periodontal breakdown, the pulpal coronal stress was barely visible for rotation, and smaller but more extended for translation, while at 8 mm of loss, it was still visible for translation. Translation, despite being quantitatively the least stressful movement (among the five tested), qualitatively displayed the most extended coronal stress along the periodontal breakdown simulations, involving (after 4 mm of bone loss) both coronal and root canal pulp. Thus, if in 1 mm of bone loss the stress exclusively involves almost the entire coronal pulp, after 4 mm, the stress extends to the radicular pulp (upper and middle thirds), while at 8 mm, the coronal involvement decreases but the radicular one stays pretty much the same. Based on the above-mentioned, it seems that translation movement is more prone (than rotation) to circulatory disturbances leading to ischemia and degenerative-resorptive risks for previously injured dental pulp (i.e., direct, and indirect pulp capping). Moreover, it seems that 4 mm of periodontal breakdown could signal a clinical stress increase with potentially ischemic and degenerative-resorptive risks for the previously traumatized/injured teeth with or without dental pulp treatments (direct-indirect pulp capping).

Intrusion, extrusion, and tipping did not show any pulpal (coronal and/or radicular) involvement.

## 4. Discussion

Our numerical analysis (the first one of its kind) totaled 720 simulations and showed that during periodontal breakdown, 2–4 N of applied orthodontic forces had very little influence over the dental pulp and the NVB of healthy intact teeth. Among the two components studied, the NVB was more affected than the dental pulp (due to its anatomical topography found in the apical third of the periodontal ligament), but with stress amounts lower than the physiological MHP (of 16 KPa). Thus, for intact healthy teeth (as our second lower premolar herein), a 1–8 mm simulated horizontal periodontal breakdown process seemed to not induce any significant ischemic and resorptive-degenerative risks for the dental pulp and the NVB (in agreement with both our [9,10,17,18,19,20,21,22] and other [12,13,14,15,16] numerical studies, but contradicting in vivo–in vitro Minch et al. [2] and Hofman et al. [15,16] reports). Among the five analyzed movements, rotation seems to be more stressful, closely followed by tipping, but with quantitative amounts of stress up to eight times (for 8 mm bone loss rotation) lower than the MHP, in line with [9,10,12,13,14,15,16,17,18,19,20,21,22] and disagreeing with [2,15,16]. It must be emphasized that from a mechanical point of view, the PDL has an absorption-dissipation role, actively limiting tissular deformations and displacements. This PDL ability is still active, since the 8 mm periodontal loss does not exceed 50% of the total height. Intrusion movement is thus naturally/physiologically restrained (as well as the extrusion, as displayed in Figure 3 and Figure 4), avoiding any ischemic risks. The stressfulness of a movement can be quantified only based on the amount of stress that is locally induced. Nevertheless, the above-mentioned refer to healthy intact tissues, where there are no morpho-pathological functional adaptations and modifications induced by trauma/injury. However, if these are present, the tissular adaptability of the pulp and the NVB is reduced, and the tissues may suffer from ischemic risks.

The above-mentioned results agree with our earlier reports [9,17] regarding the biomechanical behavior of dental pulp and NVB subjected to light orthodontic forces. Comparable reports are provided by other in vivo studies [4,5,6,7,37] and backed by the clinical principles of orthodontic treatment of Proffit et al. [44].

Our results herein (Table 2 and Figure 3 and Figure 4) are also in line with other previous numerical studies [9,10,17,18,19,20,21,22] provided by our team regarding the absorption-dissipation ability of dental and periodontal structures, reporting an extremely small percentage of the applied amounts of stress reaching the dental pulp and the NVB (also backed by common clinical data) [8,23,24,43,45,46].

Based on correlations performed among the above-mentioned, despite the apparent safe appliance of 4 N on pulp and NVB during treatment, and since the NVB is contained by the PDL, higher orthodontic forces should be used with care, as recommended by Proffit et al. [44] and other clinical and numerical reports [8,43,45,46]. It must be emphasized that since PDL has a biomechanical absorption-dissipation function, receiving most of the stresses, higher orthodontic forces could inflict local circulatory disturbances, leading to ischemia and degenerative-resorptive risks, as clinically reported [3,8,43]. On the other hand, dental living tissues have a significant capability to adapt and withstand various amounts of stress without much damage if the period of time is limited (also confirmed by clinical data) [1,2,3,8,43,45,46].

There are a few reports assessing the biomechanical behavior of dental pulp (and only in intact periodontium) [2,15,16] and none that of the NVB, except ours [9,10,17,18,19,20,21,22]. Thus, NVB correlations were used for the numerical and clinical studies of the PDL apical third (that anatomically includes the neuro-vascular bundle) in this study, as well as in our previous reports.

In the current body of research, there are general clinical reports on the biomechanical behavior of previously traumatized dental tissues [4,5,6,7,25,26,27,28,29,30,31,32,33,34,35,36,37,38,39,40,41,42] and numerical in vivo as well as in vitro reports [2,4,5,6,7,15,16,37,47,48,49,50,51,52,53,54] that can be used for indirect validation. These above-mentioned studies suggested that a clear separation between intact/healthy and previously traumatized/injured tissues’ biomechanical behavior must be performed. It must be emphasized that these traumatized tissues, despite being functionally living, always have functional limitations and scars, while their reactivity and capacity to sustain further damage is significantly diminished (also backed by common medical data) [4,5,6,7,25,26,27,28,29,30,31,32,33,34,35,36,37,38,39,40,41,42]. Thus, small circulatory disturbances that in a healthy tissue will pass unnoticed, could lead to ischemia and degenerative-resorptive risks in previously traumatized/injured tissues [4,5,6,7,25,26,27,28,29,30,31,32,33,34,35,36,37,38,39,40,41,42].

Our study is the first to study the biomechanical behavior of the dental pulp and the NVB under orthodontic movements, during periodontal breakdown, and to perform biomechanical and clinical correlations between healthy and previously injured dental tissues. The qualitative results showed significant NVB deformations during intrusion and extrusion, more visible after 4 mm of loss (Figure 3A,B and Figure 4A,B), prone to circulatory disturbances, ischemia, and degenerative-resorptive risks in previously traumatized/injured teeth and periodontium, in agreement with our previous numerical findings [9,10,17,18,19,20,21,22], those of Hofman et al. [15,16], and other studies [4,5,7,26,27,28,29,30,31,32,33,34,35,36]. Moreover, rotation (Figure 3C and Figure 4C) and translation (in particular, Figure 2E and Figure 3E) displayed visible pulpal coronal stress (proximal and vestibular sides), which involves the radicular pulp (upper and middle thirds) after 4 mm of loss, also prone to ischemic and degenerative-resorptive risks on previously traumatized pulp (direct and indirect pulp caping), in agreement with other numerical findings [9,10,17,18,19,20,21,22] and studies [4,5,6,7,23,24,25,36,37,38,39,40,41,42]. Thus, by correlating these biomechanical findings, it seems that during periodontal breakdown (especially after 4 mm of loss) 2–4 N of applied force could lead to increased ischemic risks in line with our previous numerical reports for light orthodontic forces (using a similar methodology) [9,10,17,18,19,20,21,22].

The lack of numerical studies on the dental pulp and especially the NVB is basically due to their small size and anatomical complexity, difficult to be identified on CBCT images and then be 3D-reconstructed (especially if the selection-reconstruction process was performed automatically by the reconstruction software) [3,9,17]. However, if the segmentation–reconstruction process is manually performed, the reconstruction is possible (despite the time-consuming process) [3,9,17]. The in vivo studies cannot individually study the dental pulp and NVB due to the lack of means [3,9,17]. Thus, the only method remaining is numerical analysis, which can deliver extremely accurate results as in the engineering field [9,10,17,18,19,20,21,22]. This method of studying the living tissues was adopted in the last two decades, but without adopting all the mandatory conditions related to engineering field accuracy, resulting in various reports that often contradicted clinical data [4,5,6,7,12,13,14,15,16,37,47,48,49,50,51,52,53,54].

Thus, these previous-mentioned studies reported mixed data, some in line with clinical knowledge, while others had serious accuracy problems that directly contradicted in vivo studies. Some of the dental numerical studies investigated the PDL (without even reconstructing the NVB in the PDL apical third [12,13,14,15,16]) reporting on various orthodontic movements (i.e., 1–4 N) in intact periodontium and stress amounts 2.5–5 times higher than the MHP, suggesting significant circulatory disturbances triggering ischemic, necrotic, and resorptive risks, which in clinical reality never occur. The same studies also reported data in line with clinical knowledge (e.g., rotation being the most stressful movement in intact periodontium [12,13,14]) or in disagreement with it (e.g., intrusion being the most stressful [15,16]). They also reported an optimal amount of force for rotation of 2.1–2.9 N [12,13,14], contradicting the clinical orthodontic treatment principle (e.g., Profit et al. [44]) that optimal force should be light to avoid any ischemic and necrotic risks. The qualitative stress display areas had accuracy issues (e.g., the stress reported to be concentrated only in the apical third of the PDL, while cervically it was insignificant [12,13,14,15,16]), contradicting the clinical biomechanical knowledge. Moreover, there are also reports suggesting that most clinical PDL studies suffer from accuracy issues [43,45].

In light of the above-mentioned, we must emphasize not only the need for new numerical studies investigating the dental pulp, NVB, and PDL (anatomically and functional codependent structures) following the guide-lines established in engineering fields but also that, if properly used, the method can provide accurate results (as proven by previous reports [9,10,11,17,18,19,20,21,22,57]).

The mandatory conditions needed for accurate results (as in the engineering field) are related to the anatomical accuracy, failure criteria, boundary assumptions, and sample size [9,10,11,17,18,19,20,21,22,57]. The are dental studies describing the relationship between the type of material and the failure criteria to be used (brittle [11,57], ductile), with reports on the ductile resemblance of living dental structures (proven to be correct by our previous results [9,10,17,18,19,20,21,22]). The Tresca failure criteria were reported to provide the most accurate results among the five most frequently used (Von Mises, Tresca, Maximum principal, Minimum principal, and Hydrostatic pressure) [9,10,17,18,19,20,21,22].

The boundary assumptions are related to the anisotropy, non-homogeneity, and non-linear elasticity specific to living tissues, while all previous numerical analyses [4,5,6,7,11,12,13,14,15,16,37,47,48,49,50,51,52,53,54,55,56,57,58,59,60,61] instead used the isotropy, linear-elasticity, and homogeneity without arguing the differences. However, our team’s previous studies [9,10,17,18,19,20,21,22], by analyzing these differences, reported that linear-elasticity and isotropy can be used in dental studies due to extremely small deformations and displacement (according to physical biomechanics), while the Tresca criteria were designed for non-homogenous materials.

The anatomical accuracy of the studied 3D models implies not only having the physical shape of the living tissue, but also possessing a higher number of elements and nodes, as well as a small global element size (e.g., 6.05 million tetrahedral elements, 1.07 million nodes, global element size of 0.08–0.116 mm, 40–12,731 times more elements, 4.4–1463 times more nodes than other studies [4,5,6,7,15,16,37,47,48,49,50,51,52,53,54]). The sample size of one, which is specific to the numerical studies (due to large possibilities to change the experimental conditions [11,48,49,52,55,56,57,58,59,60,61]), should be larger for enhancing accuracy, as reported by previous studies [9,10,17,18,19,20,21,22,62]. Thus, a sample size of nine (as herein) is nine times higher than any previous numerical dental study [11,48,49,52,55,56,57,58,59,60,61], confirming the increased accuracy of the report.

The numerical studies applied in dentistry and medicine cannot completely reproduce the complexity of the clinical environment (a downside of this method), thus, their results need to be correlated with clinical data (MHP and common clinical biomechanical knowledge being the most frequently used). Nevertheless, this method is the only one available at this moment allowing us to individually study each tissular component. Usually, dental numerical studies (included the study herein) use pure orthodontic movements, despite the combination and association of movements clinically occurring. Thus, it is possible that the quantitative amounts of stress reported by the numerical analyses (included the analysis herein) are clinically slightly lower. That is why more numerical studies are needed to investigate the biomechanical behavior of dental tissues, but only if the mandatory conditions needed for accurate results are followed and correlations with clinical data are performed.

## 5. Conclusions

1. During the horizontal periodontal breakdown (1–8 mm), 2–4 N of applied orthodontic force displayed similar qualitative stress distribution in all five movements, but with a quantitative increase (doubling of stress amounts for 4 N when compared with 2 N).
2. In the bone loss simulations, the highest amounts of stress were displayed at 8 mm of periodontal loss, but lower than 16 KPa of the MHP.
3. The NVB stress was higher than the pulpal stress.
4. The rotation was the most stressful, closely followed by tipping, intrusion, and extrusion.
5. A total of 4 N of applied force seems to not induce any ischemic or degenerative-resorptive risks for healthy intact teeth in up to 8 mm of periodontal breakdown.
6. Intrusion and extrusion determined the highest visible tissular deformation in NVB, with potential ischemic and resorptive-generative risks for previously traumatized/injured teeth.
7. Rotation and translation (in particular) showed the highest coronal and radicular pulpal stress with potential ischemic and resorptive-generative risks for previously injured/traumatized dental pulp (i.e., direct-indirect pulp capping).
8. It seems that 4 mm of periodontal breakdown could signal a clinical stress increase with potentially ischemic and degenerative-resorptive risks for the previously traumatized/injured teeth with or without dental pulp treatments (direct-indirect pulp capping).

## 6. Practical Implications

Few studies investigated the dental pulp (only in intact periodontium) and none the NVB; thus, the study herein provides valuable information about the biomechanical behavior of both tissues during periodontal breakdown under large forces (2–4 N) and orthodontic movements. This information is of extreme importance when conceiving the orthodontic treatment plan and assessing the short- and long-term ischemic and degenerative-resorptive risks induced by the treatment. In intact healthy teeth, the gradual horizontal periodontal breakdown seems not to induce any significant risks (the stress amounts are lower than the physiological maximum hydrostatic pressure). However, in previously injured/traumatized teeth and/or dental pulp (i.e., direct-indirect pulp capping), there are ischemic and degenerative-resorptive risks induced by larger orthodontic forces during bone loss. Thus, intrusion and extrusion movements induced higher tissular deformation of the NVB component, while rotation and translation (in particular) induced pulpal coronal and radicular stress. Moreover, after 4 mm of bone loss, these effects progressively increased along with the periodontal breakdown. Another critical issue that the report herein argues is the accuracy of the numerical analysis and the correct approach need to ensure it. Thus, from this point of view, this study is of extreme importance for both clinician and researcher.

## Figures and Tables

**Table 1:** Physical properties of materials.

Materials	Young’s Modulus, E (GPa)	Poisson Ratio, ʋ	Refs.
Enamel	80	0.33	[9,10,17,18,19,20,21,22]
Dentin/Cementum	18.6	0.31	[9,10,17,18,19,20,21,22]
Pulp and NVB	0.0021	0.45	[9,10,17,18,19,20,21,22]
PDL	0.0667	0.49	[9,10,17,18,19,20,21,22]
Cortical bone	14.5	0.323	[9,10,17,18,19,20,21,22]
Trabecular bone	1.37	0.3	[9,10,17,18,19,20,21,22]
Stainless-streel bracket (Cr-Co)	218	0.33	[9,10,17,18,19,20,21,22]

**Table 2:** Tresca shear stress average values (KPa) produced by 2-4 N orthodontic forces.

Resorption (mm)		1	2	3	4	5	6	7	8
Rotation 2 N	NVB	0.95	0.98	1.18	1.38	1.55	1.71	1.88	2.05
	% NVB	1.00	1.02	1.23	1.45	1.62	1.80	1.97	2.15
	pulp	0.09	0.09	0.11	0.13	0.14	0.16	0.17	0.19
	% pulp	1.00	0.98	1.19	1.40	1.57	1.73	1.90	2.07
4 N	NVB	1.91	1.95	2.36	2.76	3.09	3.43	3.76	4.09
	% NVB	1.00	1.02	1.23	1.45	1.62	1.80	1.97	2.15
	pulp	0.18	0.18	0.22	0.26	0.29	0.32	0.35	0.38
	% pulp	1.00	0.98	1.19	1.40	1.57	1.73	1.90	2.07
Translation 2 N	NVB	0.46	0.48	0.53	0.58	0.65	0.72	0.79	0.86
	% NVB	1.00	1.04	1.16	1.27	1.42	1.58	1.73	1.88
	pulp	0.04	0.04	0.05	0.05	0.06	0.07	0.08	0.08
	% pulp	1.00	1.13	1.26	1.39	1.59	1.78	1.97	2.17
4 N	NVB	0.92	0.96	1.06	1.17	1.31	1.45	1.58	1.72
	% NVB	1.00	1.04	1.16	1.27	1.42	1.58	1.73	1.88
	pulp	0.08	0.09	0.10	0.11	0.12	0.14	0.15	0.17
	% pulp	1.00	1.13	1.26	1.39	1.59	1.78	1.97	2.17
Tipping 2 N	NVB	0.71	0.72	0.84	0.96	1.09	1.23	1.36	1.49
	% NVB	1.00	1.03	1.19	1.36	1.55	1.74	1.93	2.11
	pulp	0.06	0.06	0.07	0.08	0.10	0.11	0.12	0.13
	% pulp	1.00	1.15	1.34	1.54	1.74	1.94	2.14	2.35
4 N	NVB	1.41	1.45	1.68	1.92	2.19	2.46	2.72	2.99
	% NVB	1.00	1.03	1.19	1.36	1.55	1.74	1.93	2.11
	pulp	0.11	0.13	0.15	0.17	0.19	0.21	0.24	0.26
	% pulp	1.00	1.15	1.34	1.54	1.74	1.94	2.14	2.35
Extrusion 2 N	NVB	0.67	0.71	0.78	0.85	1.00	1.15	1.30	1.45
	% NVB	1.00	1.06	1.16	1.26	1.49	1.71	1.93	2.15
	pulp	0.06	0.06	0.07	0.07	0.09	0.10	0.11	0.12
	% pulp	1.00	1.05	1.16	1.26	1.47	1.68	1.89	2.10
4 N	NVB	1.35	1.42	1.56	1.71	2.00	2.30	2.60	2.89
	% NVB	1.00	1.06	1.16	1.26	1.49	1.71	1.93	2.15
	pulp	0.12	0.12	0.14	0.15	0.17	0.20	0.22	0.25
	% pulp	1.00	1.05	1.16	1.26	1.47	1.68	1.89	2.10
Intrusion 2 N	NVB	0.67	0.71	0.78	0.85	1.00	1.15	1.30	1.45
	% NVB	1.00	1.06	1.16	1.26	1.49	1.71	1.93	2.15
	pulp	0.06	0.06	0.07	0.07	0.09	0.10	0.11	0.12
	% pulp	1.00	1.05	1.16	1.26	1.47	1.68	1.89	2.10
4 N	NVB	1.35	1.42	1.56	1.71	2.00	2.30	2.60	2.89
	% NVB	1.00	1.06	1.16	1.26	1.49	1.71	1.93	2.15
	pulp	0.12	0.12	0.14	0.15	0.17	0.20	0.22	0.25
	% pulp	1.00	1.05	1.16	1.26	1.47	1.68	1.89	2.10

NVB: neuro-vascular bundle stress; % NVB: no. of times of stress increase; pulp: apical third stress; % pulp: no. of times of stress increase.

## Data Availability

The data presented in this study are available on request from the corresponding author. The data are not publicly available due to privacy.
